# Activation of mTOR Ameliorates Fragile X Premutation rCGG Repeat-Mediated Neurodegeneration

**DOI:** 10.1371/journal.pone.0062572

**Published:** 2013-04-23

**Authors:** Yunting Lin, Chengyuan Tang, Hua He, Ranhui Duan

**Affiliations:** The State Key Laboratory of Medical Genetics, Xiangya Medical School, Central South University, Changsha, Hunan, China; Oslo University Hospital, Norway

## Abstract

Fragile X associated tremor/ataxia syndrome (FXTAS) is a late onset neurodegenerative disorder caused by aberrant expansion of CGG repeats in 5′ UTR of *FMR1* gene. The elevated mRNA confers a toxic gain-of-function thought to be the critical event of pathogenesis. Expressing rCGG_90_ repeats of the human *FMR1* 5′UTR in *Drosophila* is sufficient to induce neurodegeneration. Rapamycin has been demonstrated to attenuate neurotoxicity by inducing autophagy in various animal models of neurodegenerative diseases. Surprisingly, we observed rapamycin exacerbated rCGG_90_-induced neurodegenerative phenotypes through an autophagy-independent mechanism. CGG_90_ expression levels of FXTAS flies exposed to rapamycin presented no significant differences. We further demonstrated that activation of the mammalian target of rapamycin (mTOR) signaling could suppress neurodegeneration of FXTAS. These findings indicate that rapamycin will exacerbate neurodegeneration, and that enhancing autophagy is insufficient to alleviate neurotoxicity in FXTAS. Moreover, these results suggest mTOR and its downstream molecules as new therapeutic targets for FXTAS by showing significant protection against neurodegeneration.

## Introduction

Fragile X associated tremor/ataxia syndrome (FXTAS) is a neurodegenerative disease caused by aberrant expansion of CGG repeats in 5′ UTR of *FMR1* gene. The major clinical features of FXTAS are progressive intention tremor and/or ataxia accompanied by gradual behavioral deficits and cognitive decline [1], [2]. The frequency of fragile X premutation in the general population is approximately 1/813 in males, and about 30%–40% of male *FMR1* premutation carriers will ultimately exhibit some features of FXTAS by the time they are over 50 years old [3]. So far, there is no effective therapeutic intervention for FXTAS.

FXTAS results from an RNA toxic gain-of-function, and the accumulation of elevated *FMR1* mRNA is believed to be an important and proximal event in pathogenesis. Premutation-length rCGG repeats are sufficient to cause neurodegeneration. An FXTAS *Drosophila* model that ectopically expresses rCGG_90_ repeats of the human *FMR1* 5′UTR presents a series of neurodegenerative phenotypes which could imitate clinical features of FXTAS patients [4]. CGG KI mice have also shown many important aspects of FXTAS, including elevated mRNA levels, presence of intranuclear inclusions, and progressed cognitive and behavioral declines [5], [6], [7]. Neurotoxicity in FXTAS *Drosophila* and mice models reveal a highly significant association with the length and dosage of CGG tracts, and a stronger expression of rCGG_90_ has more severe consequences [4], [8].

FXTAS is a typical member of trinucleotide repeat expansion diseases (TREDs) carrying *FMR1* alleles, called premutation, with 55–200 CGG repeats. Rapamycin has been proven to protect against neurodegeneration by inducing autophagy and removing pathogenic protein aggregates in many experimental models of other TREDs, including Huntington disease (HD), spinocerebellar ataxia type 3 (SCA3) and spinal and bulbar muscular atrophy (SBMA) [9], [10], [11], [12]. As an acknowledged inhibitor of mTOR and inducer of autophagy, rapamycin has also been used in many other neurodegenerative diseases' models, such as Alzheimer's disease (AD) and Parkinson's disease (PD), to induce autophagy, to restore mTOR signaling from hyperactivity to normal levels and to decrease translation [13], [14], [15], [16]. Moreover, induction of autophagy and inhibition of the mTOR pathway have been found in human myoblasts of myotonic dystrophy type 1 (DM1), a noncoding repeat expansion RNA toxicity diseases due to gain-of-function effect of the RNA of the *DMPK* gene [17].

As the direct target of rapamycin, mTOR is a highly conserved kinase which acts as a sensor of amino acids, growth factors, energy and stress, and which coordinates cell growth, proliferation, metabolism, and cycle by regulating protein synthesis and autophagy. Sensing the intracellular stress, phosphoinositide 3-kinase (PI3K) activates AKT, a serine/threonine protein kinase also known as Protein Kinase B (PKB), which leads to a series of downstream cascades and ultimately activates mTOR. Once activated, mTOR drives the phosphorylation of downstream substrates, p70-S6 kinase (S6K) and eIF4E-binding protein 1 (4E-BP1), and regulates protein synthesis [9]. Repression of mTOR activity by nucleolar disruption restricted to dopaminergic (DA) neurons has shown progressive and differential loss of DA neurons and locomotor abnormalities that resemble PD while activation of mTOR pathway exerts neuroprotective effect [18], [19].

Here we sought to determine if FXTAS shares the common pathogenesis with other neurodegenerative diseases and could be treated with rapamycin using a *Drosophila* model. Surprisingly, we found that rapamycin could not ameliorate phenotypes, but rather led to more severe ones. Exposure to rapamycin didn't cause an increase of rCGG_90_ expression levels, and autophagy was ineffective to suppress neurotoxicity. Furthermore, activation of mTOR pathway could improve neurodegenerative phenotypes.

## Results

### Rapamycin cannot ameliorate but aggravates the neurodegenerative phenotypes in FXTAS *Drosophila* model

To determine if FXTAS shares similar pharmaceutical or genetic modifiers with other neurodegenerative disorders, we used an established *Drosophila* model of rCGG repeat induced neurodegeneration. Expressing CGG_90_ in the retina using *GMR-GAL4* drivers severely disrupted eye morphology as characterized by loss of pigmentation, neuron death (escharosis), retinal collapse, and ommatidial fusion. Expressing CGG_90_ ubiquitously using *Daughterless-GAL4* drivers shortened lifespan. Expressing CGG_90_ in the nervous system using *NRV2-GAL4* drivers led to decreased locomotion. Control flies expressing EGFP alone had no observed phenotype.

**Figure 1:**
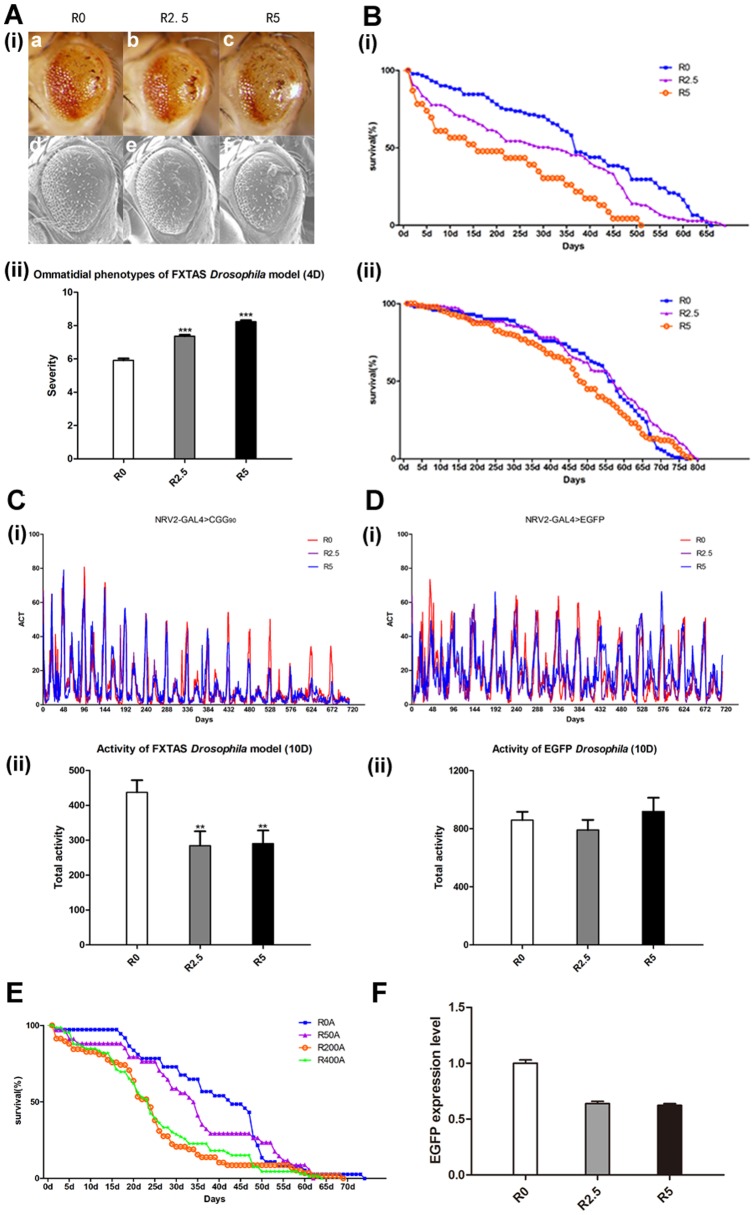
Rapamycin aggravates neurodegeneration in fly model of FXTAS. (**A**) (**i**) Rapamycin aggravates ommatidial degeneration of *GMR-GAL4>CGG_90_* (4 days old after eclosion). **a–c** LM images, **d–f** SEM images. The concentrations of rapamycin are: 0 μM (**a, d**), 2.5 μM (**b, e**), 5 μM (**c, f**). (**ii**) Quantitative analyses of eye phenotypes of *GMR-GAL4>CGG_90_* with administration of rapamycin (4 days old after eclosion). Data show mean phenotype score ± SEM (Mann-Whitney test, *** = p<0.0001). (**B**) Rapamycin shortens lifespan of *Daughterless-GAL4>CGG_90_* (**i**) while it presents no effect in *Daughterless-GAL4>EGFP* (**ii**). The concentrations of rapamycin are: 0 μM, 2.5 μM, 5 μM. (**C**) (**i**) Rapamycin decreases locomotor activity of *NRV2-GAL4>CGG_90_*. The concentrations of rapamycin are: 0 μM, 2.5 μM, 5 μM. (**ii**) Statistical analyses of locomotion of *NRV2-GAL4>CGG_90_* with rapamycin treatment (10 days old after eclosion). Data show mean locomotor activity ± SEM (Student's t-test, ** = p<0.001). (**D**) (**i**) Locomotor activity of *NRV2-GAL4>EGFP* is not altered by rapamycin. The concentrations of rapamycin are: 0 μM, 2.5 μM, 5 μM. (**ii**) Statistical analyses of locomotion of *NRV2-GAL4>EGFP* with rapamycin treatment (10 days old after eclosion). Data show mean locomotor activity ± SEM (Student's t-test, ns). (**E**) High doses of rapamycin administrated from early adulthood shortens lifespan of *Daughterless-GAL4>CGG_90_*. The concentrations of rapamycin are: 0 μM, 50 μM, 200 μM, 400 μM. (**F**) Total RNA was isolated from heads of *GMR-GAL4>CGG90* with rapamycin treatment. The concentrations of rapamycin (from column 1 to column 3) are: 0 μM, 2.5 μM, 5 μM. Rapamycin does not increase RNA levels. The mild decrease of RNA level with rapamycin treatment should be attribute to the ablation of translation by restraining phosphorylation of S6K and 4E-BP1.

To examine if rapamycin could suppress neurodegeneration in the FXTAS *Drosophila* model, we reared flies expressing retinal CGG_90_ or EGFP on food supplemented with either a DMSO control or rapamycin at doses known to mitigate neurotoxicity in other neurodegenerative diseases [10], [20]. Significant eye phenotype exacerbations of *GMR-GAL4>CGG_90_* were observed at 2.5 μM and 5 μM in repeated experiments. Rapamycin increased the appearance and affected areas of eschar, a surrogate marker for severe cell death, and loss-of-pigmentation in the retina (**Fig. 1A** **(i)**). Quantitative analyses of eye phenotypes showed significant difference between rapamycin-treated and untreated flies (**p<0.0001,** **Fig. 1A** **(ii)**). In subsequent experiments, *Daughterless-GAL4>CGG_90_* and *NRV2-GAL4>CGG_90_* were fed food containing DMSO or 2.5 μM and 5 μM rapamycin. Consistent with its role in eye phenotype, rapamycin failed to show a protective effect on lifespan and locomotion. Flies exposed to rapamycin presented dramatic reduction of lifetime (**Fig. 1B** **(i)**) and more severe locomotor activity defects (**p<0.001,** **Fig. 1C**) compared to control flies (**Fig. 1B** **(ii), D**). Effects were dose-dependent, with treatment at 5 μM rapamycin leading to more severe phenotypes than 2.5 μM. Neurodegeneration in FXTAS *Drosophila* is extremely severe and irreversible. Treatment of adult flies after eclosion with high doses of rapamycin, 50 μM, 200 μM and 400 μM, previously used to extend lifespan of flies with paraquat [16], strikingly shortened the lifespan (**Fig. 1E**) and low doses had no effect practically.

Since overexpressions of rCGG_90_ could induce neurodegeneration with dosage-dependent toxicity in *Drosophila*, we next investigated if rapamycin altered the mRNA levels of CGG_90_. Real-time PCR was used to quantify the relative level of rCGG_90_ transcripts by detecting expression of EGFP which acts as a reporter of CGG_90_. Quantitative analyses showed that treatment with rapamycin didn't increase expression levels of rCGG_90_ (**Fig. 1F**). In addition, as low doses of rapamycin have been shown to extend lifespan of *Drosophila* [21], we assessed the effects of 4 other concentrations, 1 nM, 10 nM, 100 nM and 1 μM, in longevity tests of FXTAS flies. Low doses of rapamycin could mildly extend the lifespan of control flies, but couldn't extend lifespan of FXTAS flies. Analyses of locomotion assays also strongly support the conclusion that low dosages of rapamycin were ineffective to ameliorate neuronal toxicity of FXTAS (**data not shown**). Thus, rapamycin could not ameliorate but exacerbates neurodegeneration in FXTAS *Drosophila* model without an increase of rCGG_90_ toxicity.

### Rapamycin aggravates neurodegenerative phenotypes of FXTAS flies through an autophagy-independent mechanism

**Figure 2:**
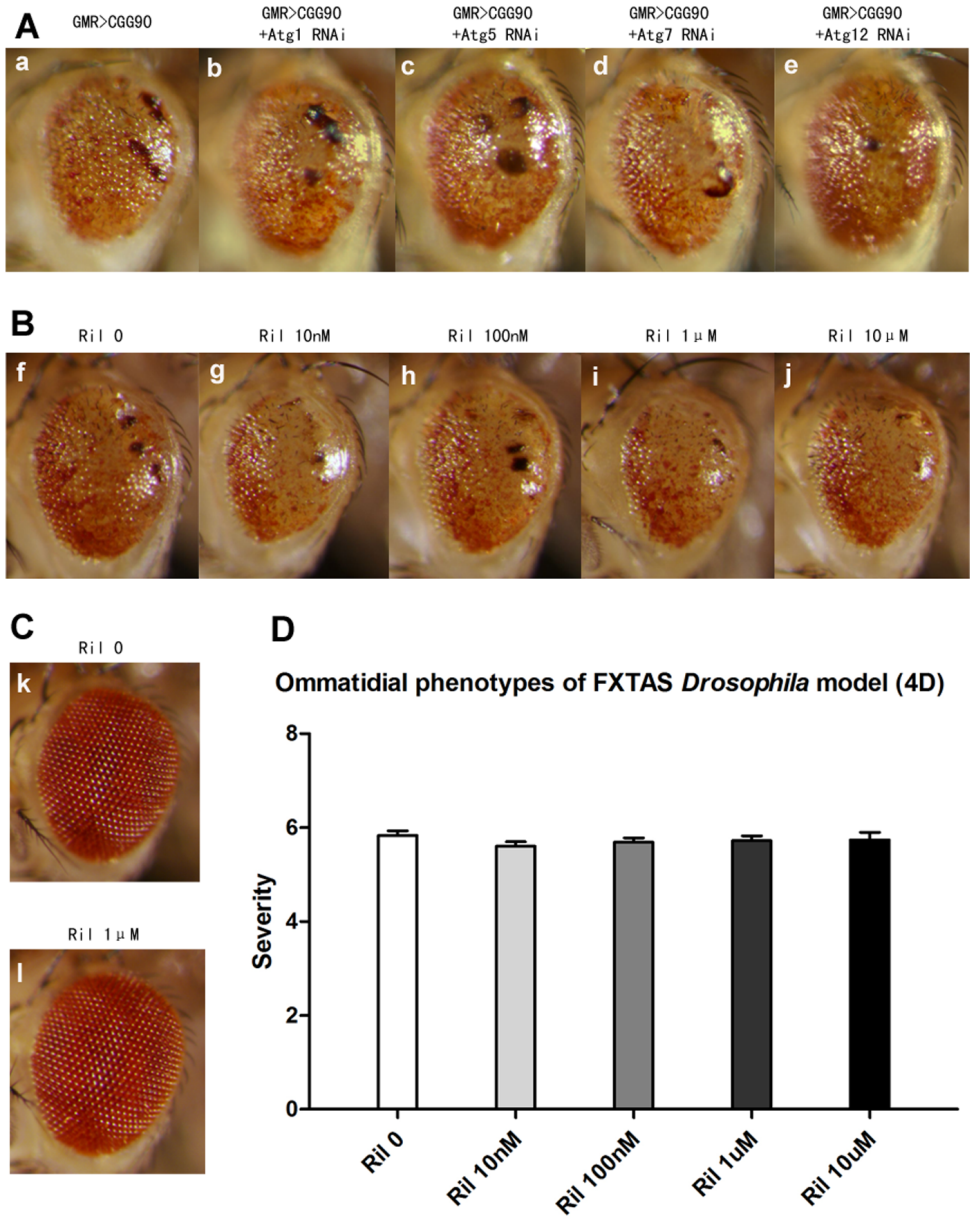
Autophagy fails to alter neurodegenerative phenotypes of FXTAS *Drosophila* model. (**A**) RNAi-mediated knockdown of Atgs doesn't alter phenotypes of *GMR-GAL4>CGG_90_* (15 days old after eclosion). **a–e** LM images. The genotypes are: *GMR-GAL4>CGG_90_* (**a**), *GMR-GAL4>CGG_90_+Atg1 RNAi* (**b**), *GMR-GAL4>CGG_90_+Atg5 RNAi* (**c**), *GMR-GAL4>CGG_90_+Atg7 RNAi* (**d**), *GMR-GAL4>CGG_90_+Atg12 RNAi* (**e**). (**B**) Rilmenidine plays an ineffective role in *GMR-GAL4>CGG_90_* (4 days old after eclosion). **f–j** LM images. The concentrations of rilmenidine are: 0 nM (**f**), 10 nM (**g**), 100 nM (**h**), 1 μM (**i**), 10 μM (**j**). (**C**) Rilmenidine also does not affect *GMR-GAL4>EGFP* (4 days old after eclosion). **k, l** LM images. The concentrations of rilmenidine are: 0 nM (**k**), 1 μM (**l**). (**D**) Quantitative analyses of eye phenotypes of *GMR-GAL4>CGG_90_* flies with administration of rilmenidine (4 days old after eclosion). Data show mean phenotype score ± SEM (Student's t-test, ns).

Acknowledged as an efficient inducer of autophagy, rapamycin has been shown to have a protective effect against many neurodegenerative diseases. To evaluate whether autophagy is involved in rapamycin's exacerbation of the CGG_90_ rough eye phenotype, we performed an RNAi-mediated knockdown of autophagy related genes (Atg 1, 5, 7 and 12 respectively) in *GMR-GAL4>CGG_90_* to attenuate the induction of autophagy. In contrast to rapamycin's aggravation of rCGG_90_-induced rough eye phenotype, co-expressing Atg RNAis showed no modification to the control phenotype (**Fig. 2A**).

Rilmenidine, an mTOR-independent autophagy-inducer, was administrated at appropriate gradient concentrations according to doses used to attenuate toxicity of polyglutamine expansions in a mouse model [22]. As expected, rilmenidine was as ineffective at modifying the CGG_90_-dependent retina degeneration as RNAi of Atgs (**Fig. 2B, D**). Control flies were also not influenced by rilmenidine (**Fig. 2C**). The above data indicate that autophagy was not involved in the exacerbation of neurodegenerative phenotypes in FXTAS flies.

### Activating mTOR can improve neurodegeneration in FXTAS

**Figure 3:**
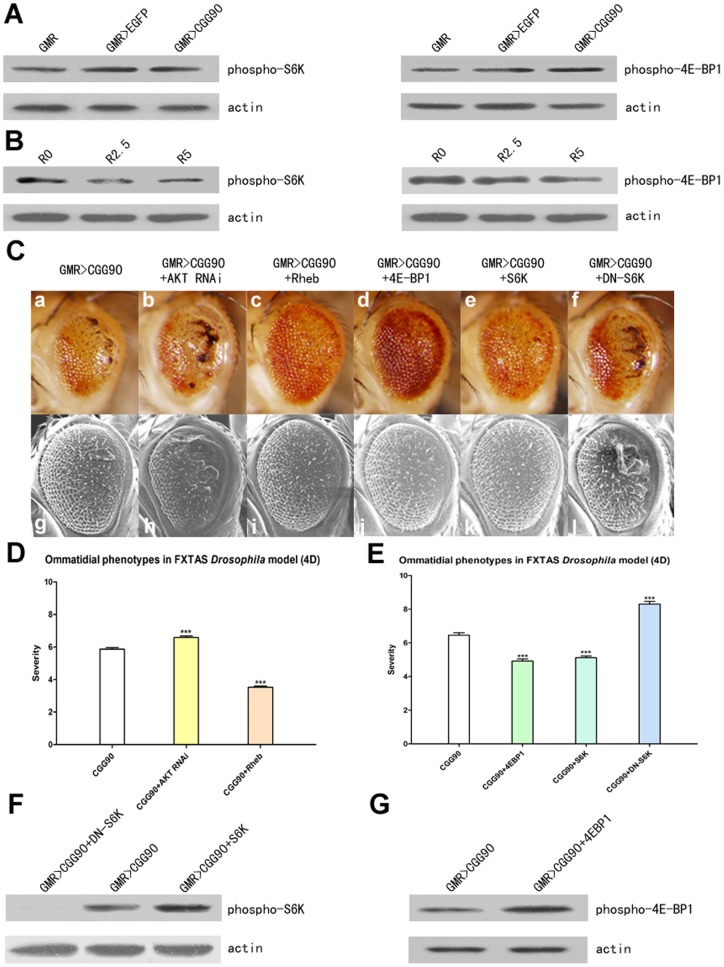
Repressing mTOR pathway exasperates neurodegeneration while activating mTOR pathway could alleviate symptoms effectively. (**A, B, F, G**) Western blotting was used to analyze mTOR activity by detecting phosphorylation of S6K and 4E-BP1. Total proteins were isolated from heads of flies. (**A**) The activity of mTOR of FXTAS flies presents no difference comparing with control flies. The genotypes (from lane 1 to lane 3) are: *GMR-GAL4/+*, *GMR-GAL4>EGFP*, *GMR-GAL4>CGG_90_*. (**B**) Rapamycin suppresses mTOR signaling and causes hypo-phosphorylation of S6K and 4E-BP1. The dosages (from lane 1 to lane 3) are: 0 μM, 2.5 μM, 5 μM. (**F**) Overexpressing S6K increases level of phosphorylated S6K while DN-S6K almost completely suppresses phosphorylation. The genotypes (from lane 1 to lane 3) are: *GMR-GAL4>CGG_90_+DN-S6K*, *GMR-GAL4>CGG_90_*, *GMR-GAL4>CGG_90_+S6K*. (G) Overexpression of 4E-BP1 increases level of phosphorylated 4E-BP1. The genotypes (from lane 1 to lane 2) are: *GMR-GAL4>CGG_90_*, *GMR-GAL4>CGG_90_+4E-BP1*. (**C**) Ommatidial phenotypes of *GMR-GAL4>CGG_90_* crossed with various modifiers. Activating mTOR ameliorate degeneration of retina while inhibiting mTOR exerts more severe phenotypes (4 day old after eclosion). **a–f** LM images, **g–l** SEM images. The genotypes are: *GMR-GAL4>CGG_90_* (**a, g**), *GMR-GAL4>CGG_90_+AKT RNAi* (**b, h**), *GMR-GAL4>CGG_90_+Rheb* (**c, i**), *GMR-GAL4>CGG_90_+4E-BP1* (**d, j**), *GMR-GAL4>CGG_90_+S6K* (**e, k**), *GMR-GAL4>CGG_90_+DN-S6K* (**f, l**). (**D, E**) Quantitative analyses of eye phenotypes of *GMR-GAL4>CGG_90_* crossed with various modifiers (4 day old after eclosion). Data show mean phenotype score ± SEM (Student's t-test and Mann-Whitney test, *** = p<0.0001).

Since rapamycin had been generally thought to be the specific inhibitor of mTOR and autophagy had been proven ineffective at altering neurodegeneration in FXTAS, here we examined the activity of mTOR pathway in FXTAS *Drosophila* model. The activation of mTOR can be measured by phosphorylation of S6K or 4E-BP1, which increases protein translation by activating a ribosomal protein rpS6 and eIF4B and by dissociating from eIF4E respectively. Western blotting analyses were performed using a phospho-Thr398-dependent S6K antibody and a phospho-Thr37/46-dependent 4E-BP1 antibody. The activity of the mTOR pathway exhibited no difference in the presence or absence of CGG_90_ (**Fig. 3A**). To determine the extent of rapamycin inhibited mTOR signaling in FXTAS flies, we measured the phosphorylation of S6K and 4E-BP1 in untreated and rapamycin-treated flies. As predicted, we observed a dose-dependent reduction in phospho-T398-S6K and phosphor-Thr37/46-4E-BP1 levels after rapamycin treatment, confirming that rapamycin suppressed mTOR signaling in FXTAS *Drosphila* (**Fig. 3B**).

To estimate if mTOR could alter neurodegenerative phenotypes, we crossed AKT RNAi and Rheb overexpressed fly lines with *GMR-GAL4>CGG_90_* as both AKT and Rheb locate upstream of mTOR in the PI3K/AKT/mTOR pathway. As expected, knockdown of AKT by RNAi in *GMR-GAL4>CGG_90_* enhanced the rCGG_90_-induced eye phenotype (**p<0.0001,** **Fig. 3C** **b, h and D**). Conversely, overexpression of Rheb conferred a protective effect against rough eye (**p<0.0001,** **Fig. 3C** **c, i and D**). These results indicate that modulating mTOR activity resulted in an alteration of neurodegenerative phenotypes of FXTAS.

To further demonstrate that activating mTOR could ameliorate eye phenotypes of FXTAS flies, we overexpressed S6K and 4E-BP1, well-described downstream targets of mTOR, in *GMR-GAL4>CGG_90_*. Overexpression of 4E-BP1 could prevent ommatidial degeneration efficiently (**p<0.0001,** **Fig. 3C** **d, i, and E**). Similarly, overexpression of S6K could suppress even severe rough eye phenotypes (**p<0.0001,** **Fig. 3C** **e, k and E**). We next crossed dominant negative S6K (DN-S6K) with *GMR-GAL4>CGG_90_*. As expected, DN-S6K expression presented as obvious retinal detriment (**p<0.0001,** **Fig. 3C** **f, l and E**). Notably, we found a substantial reduction in the viability of flies co-expressing CGG_90_ and DN-S6K to compound eye. After eclosion, over 20% *GMR-GAL4>CGG_90_+DN-S6K* flies exhibited abnormal wings which failed to expand. Western blotting analyses were used to verify the expression levels of S6K and 4E-BP1 (**Fig. 3F, G**). Based on these results, we concluded that activation of mTOR signaling could repress neurodegenerative phenotypes of FXTAS.

## Discussion

Rapamycin, a neutral macrolide with immunosuppressive properties, has been proven to extend lifespan [16], [21], [23] and to have a protective effect in many neurodegenerative diseases through induction of autophagy. As rapamycin protects against neuron death, alleviates neurotoxicity, and reduces the formation of aggregates in experimental models of other neurodegenerative disorders, we expected to see similar protective effects in FXTAS [24], [25], [26]. Unfortunately, rapamycin did not ameliorate the neurodegenerative phenotypes of FXTAS in our *Drosophila* model instead aggravating them. Recognized as an arbiter of neuronal survival and death decisions in many neurodegenerative diseases, autophagy is the most crucial cellular process involved in the clearance of redundant proteins and components [27]. Activation of autophagy has been demonstrated to mitigate neurotoxicity by promoting degradation of mutant proteins [11], [25]. Nevertheless, intriguingly, we have shown that autophagy alone has no effect on altering neurodegenerative phenotypes of FXTAS. In a previous study by Todd PK [28], overexpression of histone deacetylase 6 (HDAC6) had been shown to suppress CGG_90_ induced rough eye. Knockdown of autophagy by Atg12 RNAi had no effect on suppression of neurodegeneration by HDAC6, suggesting HDAC6 exerted protective effects by an autophagy-independent mechanism, which fits well with our findings.

In most neurodegenerative diseases, which are triggered by formation of aggregates, rapamycin or autophagy is sufficient to decrease the accumulation of mutant proteins and improve neurodegenerative phenotypes. Unlike other neurodegenerative disorders, rapamycin treatment enhanced neurodegeneration in FXTAS and activation of autophagy alone also proved to be ineffective at protecting against degeneration. These findings imply that FXTAS does not share the general pathogenic mechanism that aggregations of mutant proteins cause progressive neuronal dysfunction and loss. Therefore, we speculate that FXTAS, caused by elevated levels of mRNA, possesses unique aspects compared with other neurodegenerative diseases. It's prerequisite to distinguish FXTAS from other neurodegenerative diseases with the similar symptoms clearly in the diagnostic procedure, especially other ataxia disorders. Importantly, treatment of those patients should be cautious unless accurately diagnosed as rapamycin, or its analogues, may possibly bring about an effect opposite to the one intended.

Overexpression of fragile X premutation-length CGG repeats in mice or *Drosophila* could lead to pathological changes similar to patients, including FXTAS and fragile X-associated primary ovarian insufficiency (FXPOI) [4], [5], [6], [7], [8], [29]. In FXPOI mice model, significant reductions of phosphorylated AKT and mTOR were observed in ovaries [29]. Our findings definitely suggest that activating AKT/mTOR pathway can improve symptoms of FXTAS. Since both FXTAS and FXPOI result from toxicity of rCGG repeats, we reason that FXTAS and FXPOI share similar therapeutic intervention mechanisms and that, to some extent, the development of mTOR activators will be beneficial to them.

In FXTAS patients, the neuropathological hallmark is the presence of eosinophilic and ubiquitin-positive intranuclear inclusions, in both neuronal and astrocytic nuclei, distributed throughout the cortex and brain stem [2], [30]. *FMR1* mRNA and more than 20 protein components have been identified within the intranuclear inclusions [31], [32], [33], [34]. *FMR1* mRNA arrests those RNA binding proteins (RBPs) within inclusions and prevents them from performing normal function, which is considered the principal cause of neurotoxicity. Activation of PI3K/AKT/mTOR signaling pathway has been proven to positively regulate expression of certain protein components in inclusions, such as myelin basic protein (MBP), 2′,3′-cyclic nucleotide 3′-phosphodiesterase (CNPase) [35], [36], [37], vimentin [38], [39], [40], [41], [42], heterogeneous nuclear ribonucleoproteins (hnRNPs) [43], glial fibrillary acidic protein (GFAP) [44], HSP27 and HSP70 [45], [46]. Since those intra-inclusions proteins are positively regulated by AKT/mTOR pathway, it's rational that activation of mTOR signaling exerts remission of neurodegenerative phenotypes through increasing protein synthesis. Additionally, αB-crystallin, a small heat shock protein also identified within the intranuclear inclusions of FXTAS, has been found to increase phosphorylation of AKT and mTOR, and to activate the PI3K/AKT/mTOR signaling pathway [47]. Thus, the correlation between mTOR and protein components within conclusions needs to be further explored.

In summary, using a FXTAS *Drosophila* model carrying human *FMR1* premutation alleles, we showed that rapamycin could not ameliorate but rather exasperates rCGG_90_ induced neurodegenerative phenotypes via an autophagy-independent mechanism, and demonstrated that rapamycin didn't increase rCGG_90_-mediated neurotoxicity. Moreover, we revealed that activating mTOR signaling could suppress neurodegeneration. Consequently, our results explicitly suggest that FXTAS should be distinguished from other neurodegenerative disorders due to its unique pathogenic mechanism. Our findings also strongly support the AKT/mTOR signaling pathway as a potential therapeutic target for FXTAS, particularly the downstream substrates S6K and 4E-BP1. Further study will be necessary to identify specific downstream proteins of mTOR and to evaluate if they could restore neurodegenerative phenotypes of FXTAS.

## Materials and Methods

### Fly stocks and husbandry

Fly culture and crosses were performed on standard food according to standard procedures and raised at 25°C. *UAS-CGG_90_-EGFP* and *UAS-EGFP* used in this study were described in Jin *et al* [4]. *GAL4* were obtained from Bloomington *Drosophila* Stock Center. Fly lines of AKT/mTOR signaling were obtained from Bloomington *Drosophila* Stock Center and Vienna *Drosophila* RNAi Center.

### Drugs treatment

Rapamycin (Sangon) was dissolved in DMSO (Sigma) and added to standard food at appropriate concentrations (1 nM, 10 nM, 100 nM, 1 μM, 2.5 μM, 5 μM, 50 μM, 200 μM, 400 μM). DMSO alone was used as a control.

Rilmenidine (Sigma) was dissolved in DMSO and added to standard food at appropriate concentrations (10 nM, 100 nM, 1 μM, 10 μM). DMSO alone was used as a control.

### Scoring ommatidial degeneration

All the genotypes presented here exhibit highly uniform retinal phenotypes. We modified scoring criteria by Pandey UB [48] for proteasome impaired flies and SMBA *Drosophila* to apply quantitative analyses for FXTAS *Drosophila* in view of ommatidial phenotypes observed under light microscopy. Thousands of FXTAS flies, control flies and flies with CAG repeats were scored and our objective criteria could reflect eye phenotype truthfully. Eyes were examined and given points for the presence or absence of four phenotypes: loss-of-pigmentation, neuron death (escharosis), retinal collapse, and ommatidial fusion. Points were assigned on the following scale: 1 point was given for each phenotype present; 2 points were given if the affected area involved more than 15% of the eye; 3 points were given if the affected area involved more than 40% of the eye; and 4 points were given if the affected area involved more than 65% of the eye. Additionally, scores for neuron death (escharosis) were given twice as it was a critical feature. A higher point stands for a more severe phenotype. All the scoring analyses were performed by three persons in a double-blind method and the scoring results were completely consistent. For each genotype, over 200 4 day old flies (>300 in most cases) were examined. Comparisons were made using Student's t-test or Mann-Whitney test.

### LM and SEM

For light microscopy (LM) images, whole flies were analyzed with an OLMPUS DP72 microscope. For scanning electron microscopy (SEM) images, whole flies were analyzed with an JEOL JSM-6360-LA scanning electron microscope.

### Locomotion assay

As described previously by Qurashi A [49], homozygous progeny of 1 day old male flies were collected for locomotion assays. All the genotypes used to test locomotion were crossed with *NRV2-GAL4*. A *Drosophila* Activity Monitor (DAM) system (TriKinetics) was used to continuously monitor the locomotion. For each individual genotype, we simultaneously monitored the locomotion of at least 32 flies. Flies crossed the infrared beam and generated interruptions which were recorded as the activity of flies within 30 minutes. The readings were collected and analyzed for locomotor activity.

### RT-PCR and Real-time PCR

TRIzol (Ambion®) was used to isolate total RNA from *Drosophila*. RNA was reverse-transcribed using iScript^TM^ cDNA Synthesis Kit (BIO-RAD). Quantitative Real-time PCR was performed with the iQ^TM^ SYBR® Green Supermix (BIO-RAD) in the CFX96^TM^ Real-Time System (BIO-RAD) using EGFP-specific primers. Actin-specific primers were used as control.

### Western blotting

Protein lysis buffer (10 mM Tris-HCl, 150 mM NaCl, 30 mM EDTA, 0.5% Triton X-100, 1 mM PMSF, 1× protein inhibitors cocktail, 1× phosphatase inhibitors cocktail) was used to isolate total proteins from heads of *Drosophila*. Anti-phospho-p70 S6 Kinase (Thr398) (Cell Signaling) and anti-phospho-4E-BP1 (Thr37/46) (Cell Signaling) were used at a dilution of 1∶1000. Anti-β-actin (Abcam) (control) was used at a dilution of 1∶10000. Detection was performed with ECL (GE Healthcare).
